# A new database contains 520 studies investigating the carcinogenicity data of 238 pharmaceuticals across 14 ATC classifications

**DOI:** 10.3389/ftox.2024.1450612

**Published:** 2024-09-17

**Authors:** Panagiotis G. Karamertzanis, Martina Evangelisti, Marco Daniele Parenti, Jochen vom Brocke, Alberto Del Rio, Ingo Bichlmaier

**Affiliations:** ^1^ European Chemicals Agency, Helsinki, Finland; ^2^ Innovamol Srl, Modena, Italy; ^3^ Institute for Organic Synthesis and Photoreactivity (ISOF), National Research Council of Italy (CNR), Bologna, Italy

**Keywords:** carcinogenicity, tumour, tumorigenic potential, database, dataset, ontology, pharmaceuticals

## 1 Introduction

Recently, we compiled a new database with toxicity data from non-clinical animal studies along with human information for 528 approved drugs (Evangelisti et al., 2023). The database contains non-clinical studies for repeat-dose, carcinogenicity, developmental toxicity, and reproductive toxicity. It is made available free of charge at https://iuclid6.echa.europa.eu/us-fda-toxicity-data. The terminology used within this database has been harmonized to support further analyses, such as correlation and concordance studies. The corresponding ontology is accessible at https://github.com/innovamol/PaCCO.

The database can be used for correlation and concordance analyses (Baan et al., 2019), as well as offering insights into the tumorigenic potential of structural analogues (Alden et al., 2011) by including structural information as the original source only contained textual identifiers such as an international non-proprietary name. It facilitates the examination of species and strain sensitivities and aids in the adoption of new approach methodologies (NAMs) (Van Oosterhout et al., 1997; Reddy et al., 2010) in alignment with regulatory standards (Contrera et al., 1997).

In this research brief, our goal is to enhance data presentation for practical usage within the cancer research community. To support this, the carcinogenicity study information has been made available as Excel file (Supplementary Material).

## 2 Results and discussions

### 2.1 Data density

The database (Evangelisti et al., 2023) contains 520 studies investigating the tumorigenic potential of 238 pharmaceuticals, meaning a density of approximately two studies per drug. This finding is in line with the regulatory requirement to test in two species, usually rat and mouse.

**FIGURE 1:**
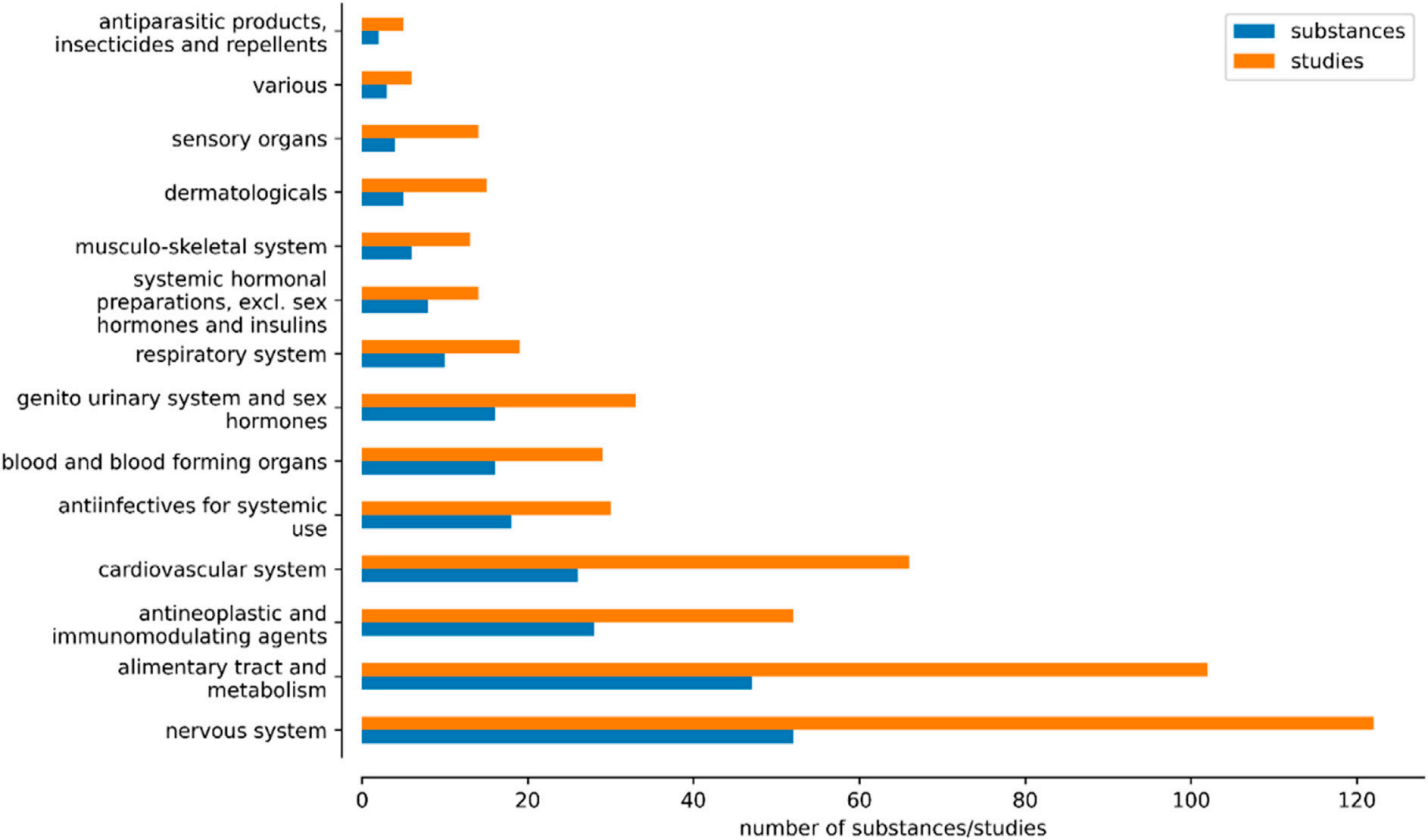
Number of substances and studies across the Anatomical Therapeutic Chemical (ATC) classifications.

The database includes approved medical drugs of 14 ATC classifications (Figure 1): 46 drugs targeting the alimentary tract and metabolism (102 studies), 18 anti-infectives for systemic use (30 studies), 28 anti-neoplastic and immunomodulating agents (54 studies), 2 antiparasitic products (5 studies), 15 drugs targeting blood and blood forming organs (27 studies), 25 cardiovascular drugs (64 studies), 5 dermatologicals (15 studies), 16 drugs targeting the genitourinary system and sex hormones (33 studies), 6 drugs acting on the musculo-skeletal system (13 studies), 50 drugs targeting the nervous system (119 studies), 12 drugs acting on the respiratory system (24 studies), 4 drugs targeting the sensory system (14 studies), 8 systemic hormonal preparations excluding sex hormones and insulins (14 studies), and 3 various drugs (6 studies).

### 2.2 Species, strain, sex specificity, and affected organs/tissues

#### 2.2.1 Species and strains

Tumorigenic potential was primarily investigated in two species: 253 studies using mice, 263 studies using rats and 4 studies reporting both species. The Chinese hamster was used in only one study, and the New Zealand White rabbit in another.

The following mouse strains were used in the indicated percentages of mouse studies: CD-1 (ca 60%), B6C3F1 (ca 10%), CB6F1 (ca 5%), other Tg (ca 4%), and NMRI (ca 4%). Other strains used in a very few studies included C57BL, SWISS, Balb/c, ICR, and specific knockout strains. In around 8% of studies, the mouse strain was not specified.

Of the 263 rat studies, 56% were conducted with Sprague-Dawley, 22% with Wistar, and 5% with Fischer 344 rats. A few other studies were performed with other albino rat strains or Long Evans rats in two studies. In approximately 7% of studies the rat strain was not specified.

The distribution of species and strains in the carcinogenicity studies of our database is consistent with the use of mouse and rat strains typically encountered when testing for tumorigenic potential for regulatory needs (Organisation for Economic Co-operation and Development, 2012).

#### 2.2.2 Sex specificity

**FIGURE 2:**
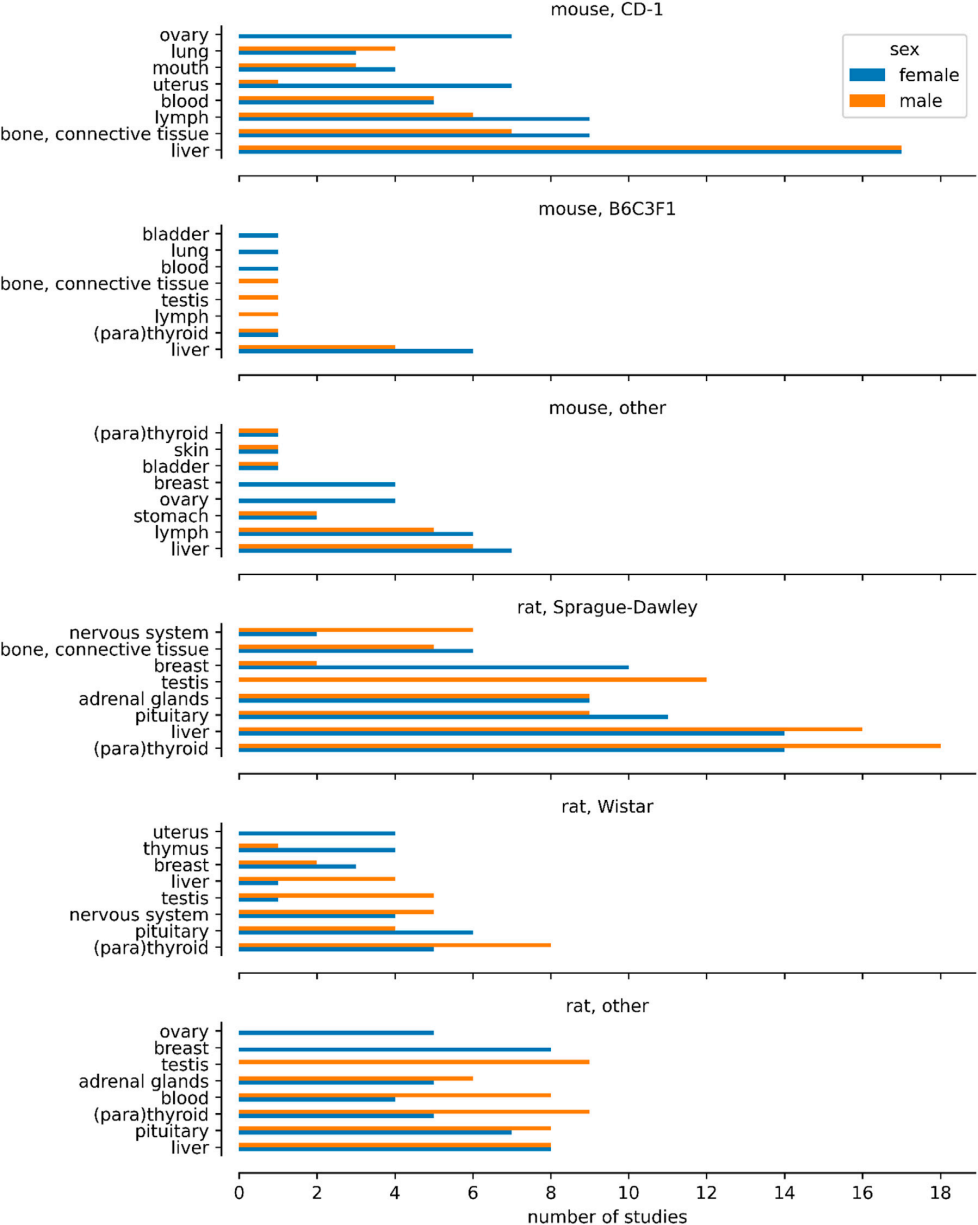
Number of studies showing affected organs and tissues in the mouse and rat strains mostly used in carcinogenicity studies of the database. All other mouse and rat strains are included in the histograms “mouse, other” and “rat, other”. In CD-1 mouse and Wistar rat, for one case the finding of tumour growth in uterus and testis was erroneously assigned to male mice and female rats, respectively. Such errors are common in large inventories of substances despite curation efforts.

Figure 2 illustrates the sex specificity of tumours targeting specific organs/tissues across the predominantly used mouse and rat strains. The figure was constructed by filtering the provided dataset so that the basis for effect corresponds to neoplastic histopathological findings, in rats or mice, and for which the sex for which the effect was reported has been provided. We then identified the two most common strains for each species and mapped all other strains to “other”. For each species and strain combination we counted the number of studies for the eight most common effects after mapping the ontology. Table 1 provides a detailed analysis, showing the incidence of various tumours by sex: in males only, females only, or in both sexes. It is important to note that not all studies were conducted in both sexes, and this is noted to ensure transparency regarding the scope of our data and to help set realistic expectations for its use.

**TABLE 1:** Substances with tumorigenic potential by affected organs/tissues and sex (No.: number, NA: not applicable).

Organ/tissue	No. Substances with findings	Males only	Both sexes	Females only
adrenal glands	26	9	10	7
blood, blood forming tissues	25	13	6	6
bone, connective tissue	28	12	5	11
Breast	27	0	4	23
intestine	3	1	0	2
Kidney	7	2	4	1
liver	59	17	27	15
thyroid + liver^a^	23	12	7	4
lung	8	4	1	3
lymph	21	3	12	6
mouth	6	1	3	2
nervous system	15	7	7	1
ovaries	15	NA	NA	15
pancreas	8	6	2	0
pituitary	32	4	17	11
skin	14	10	3	1
stomach	9	1	6	2
testes	31	31	NA	NA
thymus	7	1	2	4
thyroid/parathyroid^b^	44	17	21	6
urinary bladder	2	0	2	0
uterus	16	NA	NA	16

^a^ Observed thyroid hyperplasia, adenoma, and/or carcinoma in presence of liver findings. All findings in the 23 substances are from rat studies. No such overlap was observed in mouse studies because the database only contains 3 mouse studies with neoplastic changes in the thyroid in the absence of liver effects.

^b^ Tumorigenic potential in parathyroid was observed for 11 substances (2 in males only, 9 in both sexes).

#### 2.2.3 Affected organs and tissues

**FIGURE 3:**
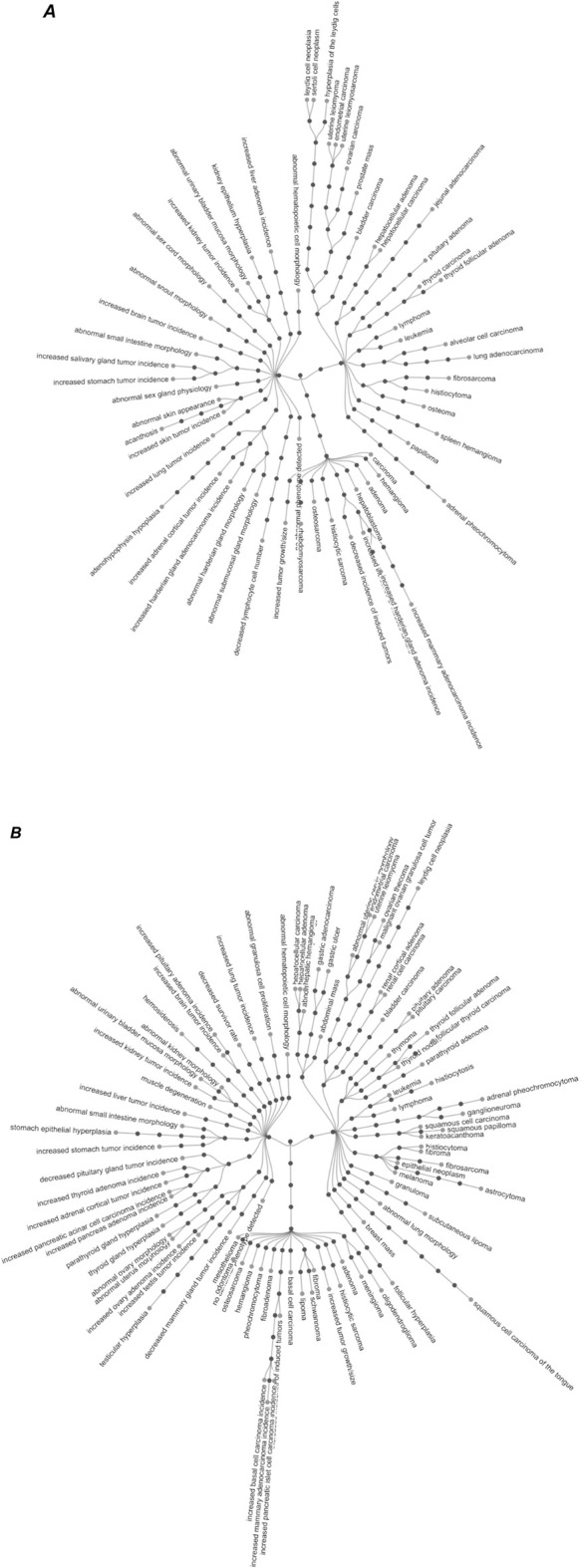
Visualization of the ontology terms associated with neoplastic histopathological effects in in mice **(A)** and rats **(B)**. Only the leaf ontology terms that have been associated with the effect level are labelled. The remaining of the ontology terms in the ontology hierarchy are only shown as filled circles to illustrate the ontology architecture. Both visualizations are provided as high-resolution png files in the Supplementary Material for improved readability. In a few instances, the same leaf term was present in more than one ontology hierarchy. For better readability, only one path for such leaf terms is depicted. The interested reader can visualize the full ontological structure in the owl of PaCCO (https://github.com/innovamol/PaCCO) while the path in a human-readable format is provided in the supplementary Excel file (column: ontology term hierarchy). Supplementary Material also provide high-resolution images.

Figure 3 shows the ontology terms associated with the effect levels plotted in Figure 2. We note that the same ontology term can appear in different hierarchies in the ontology, in which case it is shown only once.

In the genitourinary system, 7 pharmaceuticals showed tumorigenic potential in the kidneys (2 in males only, 4 in both sexes, and 1 in females only). Regarding hepatobiliary organs and tissues, in the liver, 59 pharmaceuticals induced tumour growth (17 in males only, 27 in both sexes, 15 in females only).

In reproductive and endocrine organs/tissues, 26 drugs impacted the adrenal glands (9 in males only, 10 in both sexes, 7 in females only), while 27 influenced mammary tissues (none in males only, 4 in both sexes, 23 in females only). In addition, 44 substances affected the thyroid/parathyroid (17 in males only, 21 in both sexes, and 6 in females only), with parathyroid effects observed in 11 substances (2 in males only and 9 in both sexes). Tumours also developed in the ovaries (15 substances) and the uterus (16 substances). The pituitary was affected by 32 pharmaceuticals (4 in males only, 17 in both sexes, 11 in females only), and 31 substances impacted the testes.

For the immune system, 21 pharmaceuticals promoted growth in lymphatic tissues (3 in males only, 12 in both sexes, 6 in females only), and 7 affected the thymus (1 in males only, 2 in both sexes, 4 in females only).

Table 1 includes additional data on pharmaceuticals with tumorigenic potential affecting blood constituents, blood-forming tissues, bone, connective tissues, intestine, lungs, pancreas, skin, stomach, and urinary bladder.

For 23 NDAs, tumorigenic potential in rat thyroid glands (hyperplasia, adenoma, and/or carcinoma) was observed in conjunction with liver effects (e.g., increased liver weight, liver hypertrophy, and/or hyperplasia). This finding aligns with the established understanding that neoplastic changes in the thyroid of rodents can occur as a secondary consequence of liver effects altering the metabolism of thyroid hormones (Bartsch et al., 2018).

#### 2.2.4 Concern for tumorigenic potential stemming from repeatdose toxicity studies

Our database (Evangelisti et al., 2023) includes information from repeat-dose toxicity studies, which are valuable for identifying potential concerns regarding tumorigenic potential (the original source files are attached to the IUCLID dossiers of the database (Evangelisti et al., 2023; IUCLIDa, 2024); IUCLID stands for International Uniform ChemicaL Information Database (IUCLIDb, 2024)). The distribution of species and strains in our database does not necessarily reflect ICH guidance (S1B) (European Medicines Agency, 2022), as many data are linked to experimental studies performed before these guidelines were established. However, it is important to consider these guidelines as published by the EMA can also be effectively used for comparison and context (European Medicines Agency, 2022). Indeed, sub-acute, sub-chronic and chronic repeat-dose toxicity studies reveal morphological changes, such as the presence of poorly differentiated or undifferentiated cells and hyperplasia in tissues and organs, signalling potential carcinogenic concerns. Moreover, these studies contribute to a weight of evidence approach, supporting conclusions about tumorigenic potential.

For example, our database lists 679 ‘hyperplasia’ events in different organs and tissues (for example, adrenal glands, bone marrow, gastric glands, mammary glands, liver, and thymus) among 65,403 reported effects across the 2,270 oral repeat-dose toxicity studies (Evangelisti et al., 2023). Another valuable example can be represented by liver and thyroid tumorigenesis data. The database reports 581 liver effects (for example, increased liver weight, enlarged liver, liver inflammation, abnormal liver morphology) and 184 thyroid effects (for example, thyroid gland hyperplasia, thyroid carcinoma, thyroid follicular adenoma). All carcinogenicity studies on more than 50 compounds found a total of 3,365 effects in the ATC class connected to the nervous system, which is one of the most represented categories. Out of these, 93 individual effects are classified as histopathology neoplastic (duplicates excluded). The most common type of tumour found is hepatocellular adenoma.

## Author’s note

The carcinogenicity study information is available in Excel form as Supplementary Material. The dataset has been created by extracting the pre-clinical carcinogenicity study information from the IUCLID dossiers (Evangelisti et al., 2023; Bartsch et al., 2018) that were compiled from the original pharmacological reviews provided by the US Food and Drug Administration (USFDA) (U.S. Food and Drug Administration). The dataset contains the following information in the corresponding color-coded column groups:

- substance name, CAS number, IUPAC name, application number (NDA) and ATC anatomical class
- administrative information for the carcinogenicity study, such as the dossier and endpoint study record UUID in the IUCLID database; the column esr_data contains all available information for the study as a json string, to facilitate the extraction of additional information other than what has already been included in columns; columns that contains “(code)” in the name contain the integer code of a IUCLID pick list entry and are accompanied by a column that has “(text)” in the name in which the integer code has been mapped to the corresponding IUCLID phrase
- species, strain, route of administration, duration and frequency of treatment, and doses
- description of the incidence and severity of effects that includes a qualitative description of the observed effects, and if the data allows, whether they are adverse, non-adverse, reversible, or irreversible
- details on results, carcinogenic effects and potential, conclusions and the study executive summary
- effect levels, i.e., the exposure level that corresponds to a quantified level of effects, e.g., NOAEL (No Observed Adverse Effect Level) or LOAEL (Lowest Observed Adverse Effect Level); the dataset contains one row per effect level, i.e., there may be more rows than the number of unique studies that can be identified by the column combination UUID (dossier) and UUID (parent)
- ontology information (Evangelisti et al., 2023) with the ontology ID and label assigned to the effect level; we also include the parent ontological identifier (parent ID) and the ancestral path of labels, beginning with the current term and extending upward to the root of the hierarchy.

## Data Availability

The datasets presented in this study can be found in online repositories. The names of the repository/repositories and accession number(s) can be found in the article/Supplementary Material.

## Abbreviations

ATC, Anatomical therapeutic chemical classification system; ID, Identifier; IUCLID, International uniform chemical information database; IUPAC, International Union of Pure and Applied Chemistry; LOAEL, Lowest observed adverse effect level; NDA, New drug application; NAM, New approach methodologies; NOAEL, No observed adverse effect level; US FDA, US Food and Drug Administration; UUID, Universally unique identifier.
