# Supplementary figures and images for: A new database contains 520 studies investigating the carcinogenicity data of 238 pharmaceuticals across 14 ATC classifications
Source: Front Toxicol. 2024 Sep 17;6:1450612. doi: 10.3389/ftox.2024.1450612 (PMC11442431; doi:10.3389/ftox.2024.1450612)

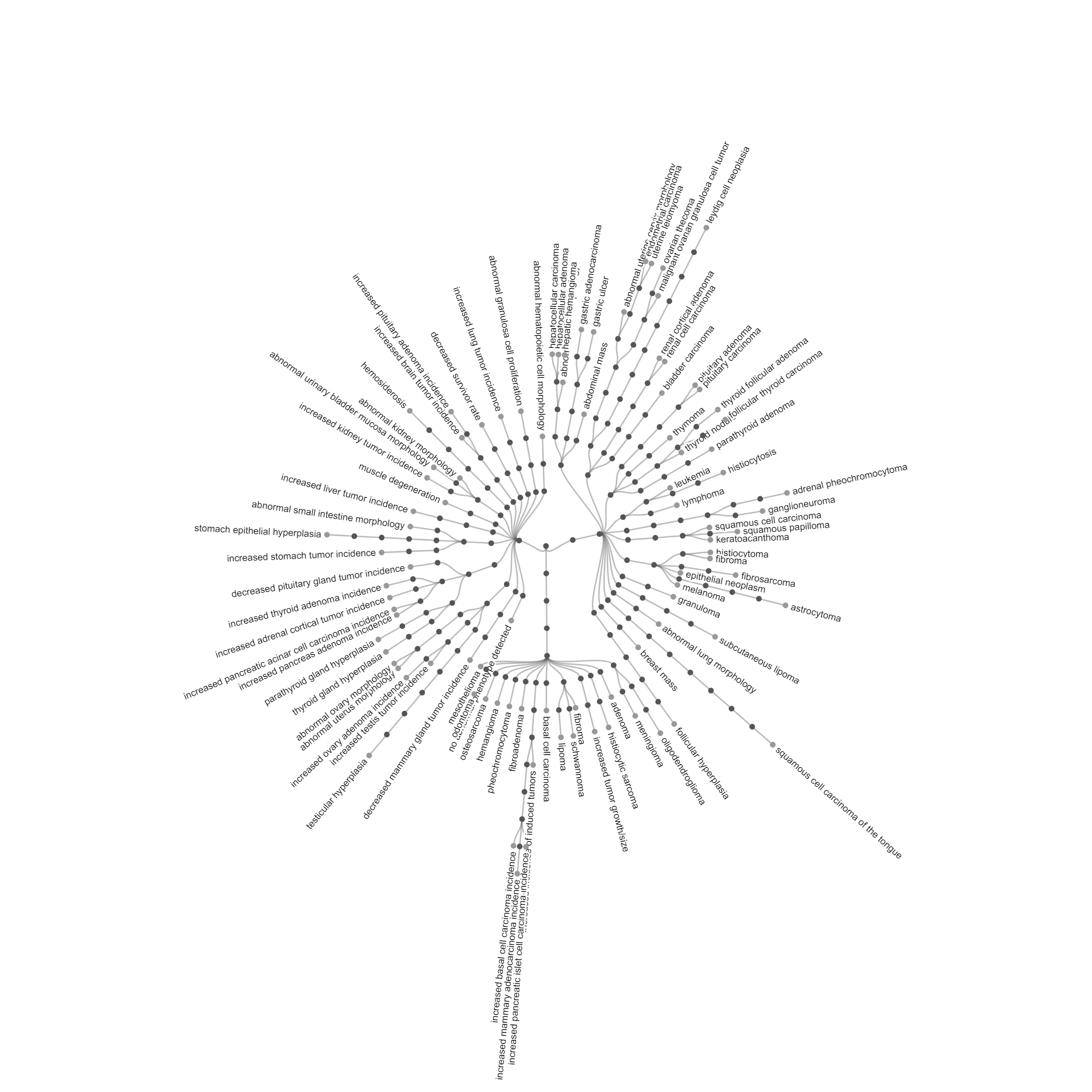

Supplement: Supplementary file 1 [file Image2.png]

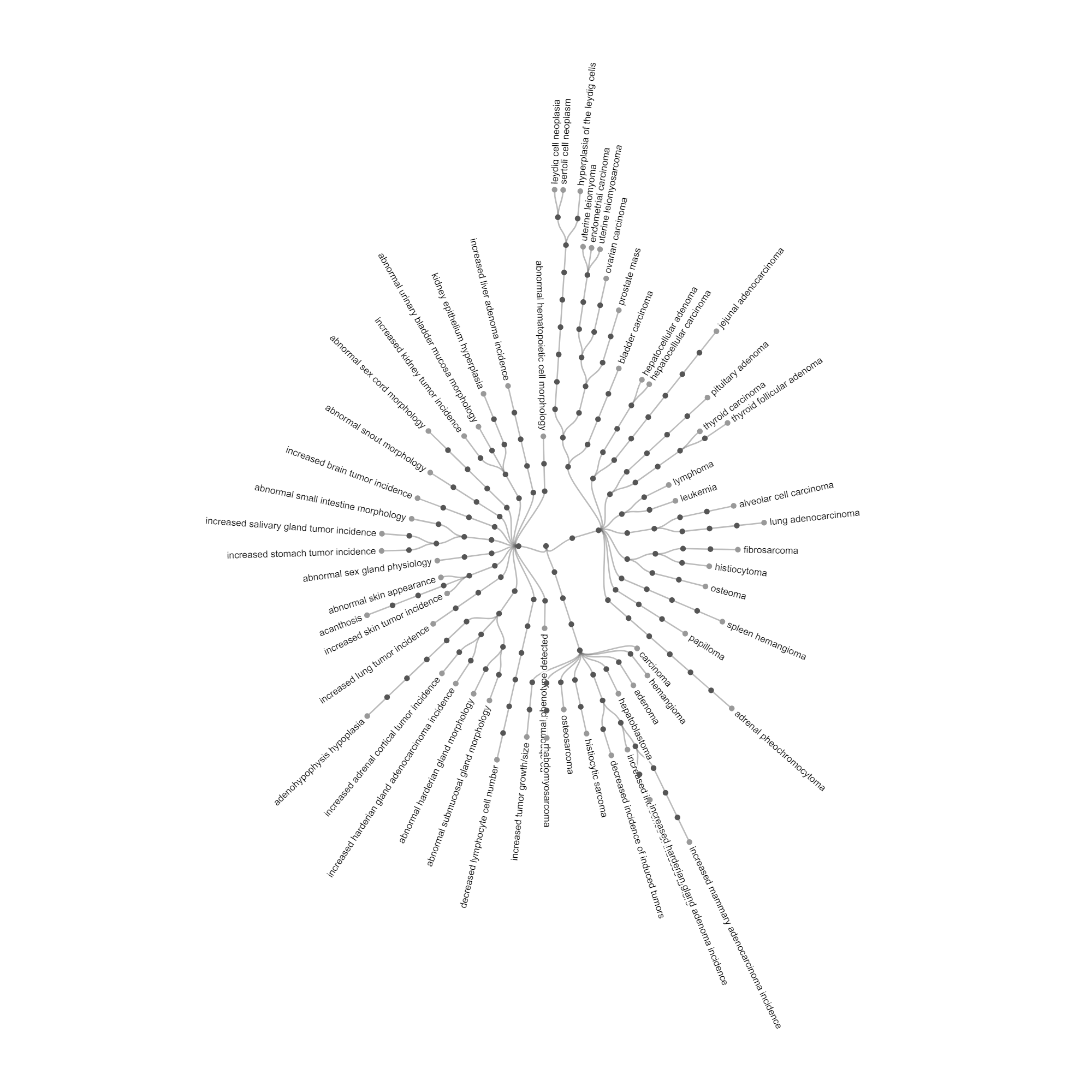

Supplement: Supplementary file 3 [file Image1.png]
